# Germination of *Bacillus* spores by LiCl

**DOI:** 10.1128/jb.00510-24

**Published:** 2025-02-27

**Authors:** James Wicander, John Gorsuch, Longjiao Chen, Rebecca Caldbeck, George Korza, Stanley Brul, Graham Christie, Peter Setlow

**Affiliations:** 1 Department of Molecular Biology and Biophysics, UConn Health 705913 , Farmington, Connecticut, USA; 2 BiOWiSH Technologies, Cincinnati, Ohio, USA; 3 Molecular Biology & Microbial Food Safety, Swammerdam Institute for Life Sciences, University of Amsterdam 215691 , Amsterdam, North Holland, Netherlands; 4 Department of Chemical Engineering and Biotechnology, University of Cambridge 151957 , Cambridge, UK; The Ohio State University, Columbus, Ohio, USA

**Keywords:** spores, *Bacillus subtilis*, germination, *Bacillus cereus*

## Abstract

Spores of *Bacillus subtilis* have been found to germinate when incubated with LiCl, but not with other monovalent or divalent metal cations. *Bacillus megaterium* spores also germinated with LiCl, but *B. cereus* spores did not. In *B. subtilis*, the LiCl germination was via the activation of spores’ GerA germinant receptor (GR), and in *B. megaterium,* it was the GerU GR. Notably, LiCl germination was much slower than normal physiological germinant triggered GR germination. In *B. subtilis* spores, rates of LiCl germination were increased in spores with a more fluid IM and decreased in spores with a less fluid IM. Analyses of the GerA germinant binding site suggested that Li^+^ could bind in a specific site in the *B. subtilis* GerAB subunit where normally a Na^+^ likely binds. Importantly, NaCl strongly inhibited LiCl germination of *B. subtilis* spores, much more so than the larger cation in KCl, although neither salt inhibited L-alanine germination via the GerA GR. These findings increase the understanding of features of mechanisms of germination of *Bacillus* spores.

**IMPORTANCE:**

The ability of some bacteria to form spores upon nutrient starvation confers properties of metabolic dormancy and enhanced resistance to environmental stressors that would otherwise kill vegetative cells. Since spore-forming bacteria include several notable pathogens and economically significant spoilage organisms, insight into how spores are stimulated to germinate and form new vegetative cells is important. Here, we reveal that relatively high concentrations of the inorganic salt lithium chloride trigger the germination of *Bacillus subtilis* and *Bacillus megaterium* spores by stimulating one of the spores of each species cohort of nutrient germinant receptors. This is significant since novel germinants and increased knowledge of the germination process should provide opportunities for improved control of spores in healthcare, food, and environmental sectors.

## INTRODUCTION

Spores of *Bacillus* species are dormant, resistant, and survive for long periods but can rapidly come back to life in germination (1–3). The latter process is triggered by physiological germinants, commonly molecules such as amino acids, sugars, or purine nucleosides, whose presence presumably signals that spores’ environment is conducive for cell growth. The germinants interact with 3-subunit germinant receptors (GRs) present in the spore’s inner membrane (IM), which are germinant-activated ion channels (4). GR activation also leads to rapid excretion of the spore core’s large depot (~20% of dry wt) of Ca^2+^ in a 1:1 chelate with dipicolinic acid (CaDPA) via IM channels comprised of SpoVA proteins. CaDPA release then triggers completion of spore germination by activating hydrolysis of spores’ peptidoglycan cortex by either of two cortex-lytic enzymes (CLEs), CwlJ and SleB, all this with minimal if any requirement for ATP. Notably, there is much-applied interest in spore germination, as when spores germinate, they are relatively easy to kill (3).

The model spore former *Bacillus subtilis* has five operons, each encoding a GR’s A, B, and C subunits (1). The GerA GR responds to L-alanine or L-valine, whereas the GerB and GerK GRs cooperatively trigger germination with a mixture of L-asparagine, glucose, fructose, and K^+^ (AGFK); the other two GRs have no known germinants, although at least one is present in spores IM (1, 5). In addition, at least the GerA, GerB, and GerK GRs are in an IM complex termed the germinosome in which the IM GerD protein acts as a scaffold (1, 6). There are one or two germinosomes in *B. subtilis* spores, and germinosome formation increases spore germination rates ≥ 10-fold (3, 6, 7). GRs can also be activated by hydrostatic pressures of ~150 megaPascals. In addition, the GR-independent germinants dodecylamine and high levels of CaDPA act on other components of the germination apparatus, including the multi-SpoVA protein channel for CaDPA release (dodecylamine) or the CLE CwlJ (CaDPA) (1). However, the latter two germinants likely have no physiological relevance, given the spores are unlikely to encounter activating concentrations of either chemical in the natural environment.

As noted above for AGFK germination, there are a number of examples of stimulatory effects of monovalent cations on spore germination (1, 8–10). There is also one well-documented example of spore germination in response to an inorganic salt alone. This is the rapid germination of *Bacillus megaterium* spores by KBr (9), which appears to activate the GerU GR that is also activated by glucose, proline, and, to a lesser extent, leucine (11). Surprisingly, in an analysis of the effects of lithium ions on endospore viability during storage, LiCl was found to cause a moderately rapid loss in viability of spores of a *B. subtilis* strain (Setlow, P; personal communication). This led to testing of the effects of LiCl on *B. subtilis* spores, and LiCl was observed to trigger these spores’ germination, a first for *B. subtilis* spores. These observations led to the studies reported in this communication where we wanted to determine how LiCl triggers *B. subtilis* spore germination and examine the LiCl germination of spores of several other *Bacillus* species. Notable findings in our work were: (i) LiCl germination of *Bacillus* spores was via the GerA GR in *B. subtilis* and GerUV in *Bacillus megaterium*; (ii) there were major effects of IM fluidity on rates of *B. subtilis* spore germination with LiCl; (iii) one mutation in the GerAB subunit, the likely germinant binding subunit (12), that abolished L-alanine germination also abolished LiCl germination; and (iv) NaCl strongly inhibited LiCl germination but KCl much less so, although neither salt inhibited L-alanine germination via GerA. These results as well as GerAB structure modeling suggest where Li^+1^ may bind in the GerA GR to trigger spore germination.

## RESULTS

### *B. subtilis* spore germination with LiCl

**Fig 1:**
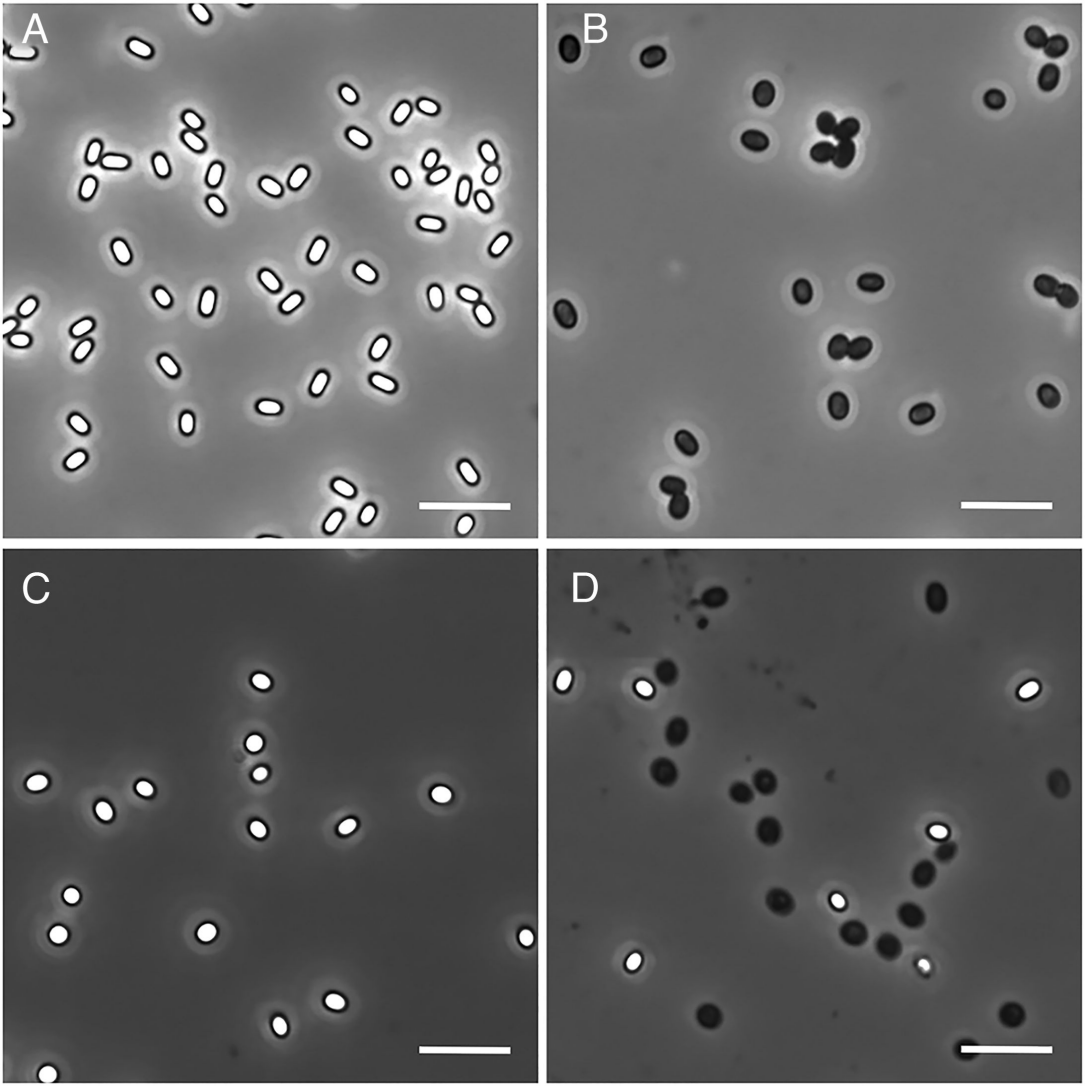
Phase contrast microscopy images of *B. subtilis* and *B. megaterium* spores incubated in LiCl. Key: *B. subtilis* spores incubated at 40°C for 48 h in 25 mM K-Hepes buffer, pH 7.4, containing (**A**) 0 mM LiCl or (**B**) 500 mM LiCl; *B. megaterium* PV361 pHT-*gerUV* spores incubated for 8 days at 30°C in 5 mM Tris-HCl, pH 7.5, containing (**C**) 0 mM LiCl or (**D**) 250 mM LiCl. Spores were imaged after 48 h incubation. Scale bar = 5 µm.

An approximate 70% loss in *B. subtilis* wild-type spore viability in 2 weeks, seen when spores were incubated with LiCl at 40°C, was surprising. A possible explanation is that LiCl was triggering spore germination, since if spores germinate in a relatively nutrient-free environment the emergent cells slowly die due to the lack of nutrients (13). To test if LiCl was indeed triggering spore germination, spores of our standard wild-type (wt) *B. subtilis* strain, PS832, were germinated with 10–500 mM LiCl at 40°C, and percentages of spore germination were determined (Table 1). Although germination with 10 mM LiCl was slow, the rate of germination increased as the LiCl concentration was incrementally increased up to 500 mM, and germination at 40°C was almost complete in 2 days with ≥100 mM LiCl. Germination in this experiment was measured by phase contrast microscopy, and the spores became as phase dark as L-valine germinated spores; this indicates that not only did LiCl trigger CaDPA release, which contributes partially to the loss of phase brightness, but that CaDPA release then triggered cortex hydrolysis, which results in phase dark spores (Fig. 1). In contrast, spores that release CaDPA but without cortex lysis are only slightly dimmer than dormant spores (1, 14).

**TABLE 1:** Germination of *B. subtilis* spores with various monovalent and divalent cations^*a*^

Salts tested	Concentration (mM)	Germination (%) after incubation at 40°C for			
		0d	1d	2d	5d
NaCl, KCl, or CsCl	100	0	1	0	1
MgCl_2_	100	0	1	2	2
CaCl_2_	100	0	4	3	5
LiCl	10	0	35	89	100
LiCl	100	0	70	100	^*b*^
LiCl^*c*^	100	0	0	0	^*b*^
LiCl	250	0	85	100	^*b*^
LiCl	500	0	98	100	^*b*^
LiBr	10	0	41	92	^*b*^
LiBr	100	0	85	^*b*^	^*b*^
Li Acetate	100	0	80	^*b*^	^*b*^
Li_2_SO_4_	100	0	85	^*b*^	^*b*^
LiCl (PS4498)	10	0	0	^*b*^	^*b*^
LiCl (PS4498)	100	0	1	^*b*^	^*b*^

^*a*^ Spores of all strains tested were wt PS832 except for PS4498 lacking all GRs and were incubated at 40°C, unless otherwise noted, in 25 mM K-Hepes (pH 7.4) with ~2x10^8^ spores/mL, and at various times, aliquots were examined by phase contrast microscopy, and ~ 100 spores were scored as phase bright (dormant) or phase dark (germinated).

^*b*^ Not analyzed.

^*c*^ Incubated at 50°C.

Germination in these experiments was due to the Li^+^ ion, as other monovalent chloride salts at 100 mM gave no germination, nor did high concentrations of several divalent cations. That fact that it was the Li^+^ that was triggering germination, and not Cl^-^, was shown by the similar germination of *B. subtilis* spores by several other Li^+^ salts (Table 1). Notably, there was no spore germination with 100 mM LiCl at 50°C (Table 1), suggesting that the LiCl germination seen at 40°C involves the normal *B. subtilis* GR-dependent germination pathway, as L-valine germination via the GerA GR was also abolished at 50°C (Table 1 and data not shown).

### Mechanism of *B. subtilis* spore germination with LiCl

**Fig 2:**
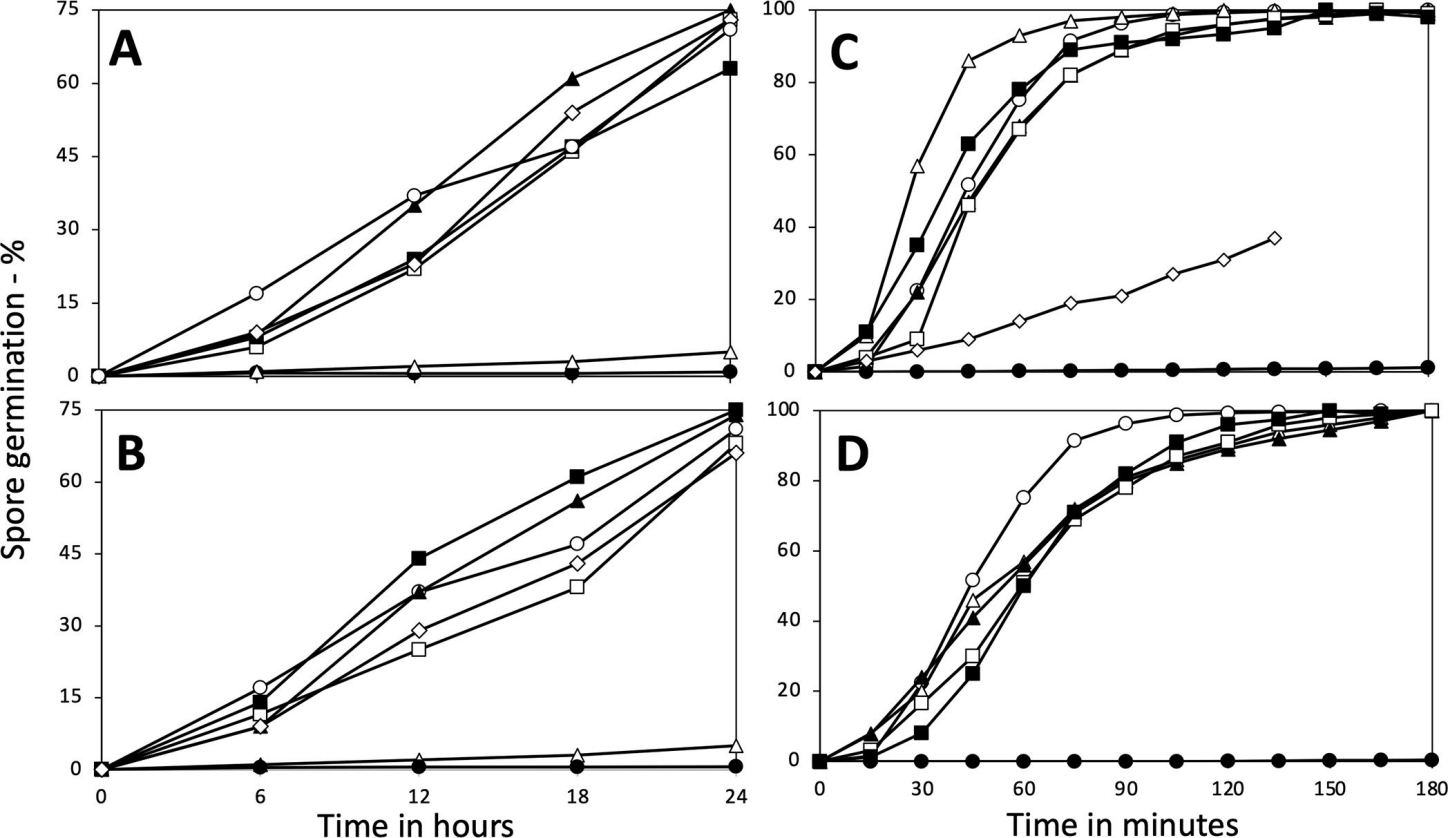
Analysis of effects of deletion mutations with (**A and C**) or without (**B and D**) an antibiotic resistance marker, on spore germination with LiCl (**A and B**) or L-valine (**C and D**). Spores of various strains were germinated in duplicate with LiCl or L-valine, and the percentages of spore germination were determined by measurement of dipicolinic acid release as described in Methods. Presented data are average values where variance in individual data points was <10%. Symbols of the strains used are: (A) ◯, PS832 (wt) (this strain has no antibiotic marker); ⬤, PS4492 (*ΔgerA*); △, FB62 (*ΔgerD*); ▲, PS4584 (*ΔgerKA*); □, PS4585 (*ΔgerBA*); ■, PS4586 (*ΔyndD*); and ◇, PS4587 (*ΔyfkQ*); (**B**) ◯, PS832 (wt); ⬤, PS4589 (*ΔgerA*); △, FB62 (*ΔgerD*; this strain does have an antibiotic marker); ▲, PS4590 (*ΔgerKA*); □, PS4591 (*ΔgerBA*); ■, PS4592 (*ΔyndD*); and ◇, PS4593 (*ΔyfkQ*); (**C**) ◯, PS832 (wt) (this strain has no antibiotic marker); ⬤, PS4492 (*ΔgerA*); ◇, FB62 (*ΔgerD*); △, PS4584 (*ΔgerKA*); ▲, PS4585 (*ΔgerBA*); □, PS4586 (*ΔyndD*); and ■, PS4587 (*ΔyfkQ*); (**D**) ◯, PS832 (wt); ⬤, PS4589 (Δ*gerA*); △, PS4590 (*ΔgerKA*); ▲, PS4591 (*ΔgerBA*); □, PS4592 (*ΔyndD*); and ■, PS4593 (*ΔyfkQ*).

To determine the mechanism of the LiCl germination of *B. subtilis* spores, the germination of multiple isogenic strains with mutations in germination proteins was examined (Table 1; Fig. 2A). Notably, the LiCl germination seen with PS832 spores was eliminated in PS4498 spores lacking all five GRs (Table 1), indicating that one or more GRs was giving rise to the LiCl germination. Examination of the germination of spores lacking single GRs (Fig. 2A) showed that the absence of the GerA GR abolished LiCl germination, whereas the absence of either GRs with no known germinants (YfkQRT or YndDEF) or the GerB or GerK GRs had only small effects on rates of LiCl germination. In addition, all the GR mutant spores, except the one lacking GerA reached ~75% germination in 24 h, as did wt spores (Fig. 2A). Loss of the GerD protein that forms the scaffold for germinosome formation also greatly decreased the rate of LiCl germination (Fig. 2A), as also seen for GR-dependent germination (6). Notably, the L-valine germination of the parental wild-type strain via the GerA GR (Fig. 2C) was much more rapid than the LiCl germination. However, all strains except the one lacking the GerA GR exhibited relatively similar rates of L-valine germination, whereas the absence of GerD slowed the rate of L-valine germination considerably, as expected (1, 6).

One potential confounding concern about the results in Fig. 2A and C was that all strains used except the wt strain, PS832, carried one or more antibiotic resistance markers replacing the gene for the absent GR. Notably, recent work (15) has indicated that the presence of a gene or plasmid carrying antibiotic resistance markers can lead to significant effects on the *Bacillus cereus* spore proteome; this was the case although antibiotics were not present during cell growth or sporulation, as was the case with spore preparation in the current work. Given these latter reports, we also prepared GR-less strains with no antibiotic markers and again measured LiCl and L-valine germination of the various strains (Fig. 2B and D). Again, there was general similarity between all strains except those lacking GerA in their LiCl and L-valine-mediated germination. Thus, it seems very unlikely that the presence of the antibiotic markers in our constructs had significant effects on rates of GR-dependent LiCl or L-valine spore germination.

**Fig 3:**
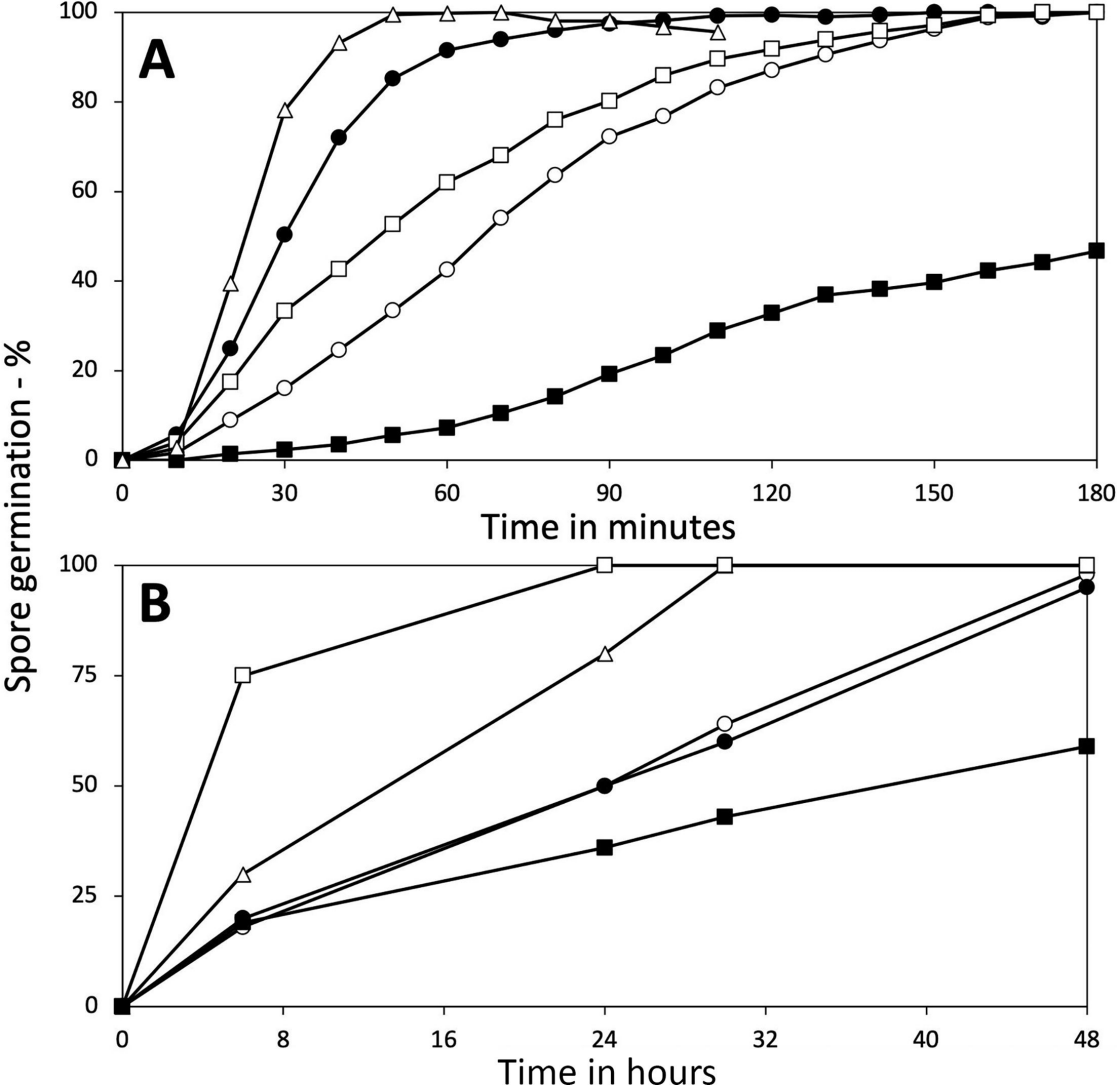
Effects of various mutations or heat activation on *B. subtilis* spore germination with (A) L-valine or (B) LiCl. Purified spores of various *B. subtilis* strains were germinated in duplicate with or without a heat shock as described in Methods with either: (A) L-valine, and percent germination was determined by measuring dipicolinic acid release, and the results from duplicate germinations were averaged as described in Methods, or (B) 100 mM LiCl, and the levels of germination were determined at various times by phase contrast microscopy as described in Methods. Variance in individual data points was <9%. All strains are isogenic with strain PS832, except PS4461 and PS4462 which are isogenic with each other. The symbols in (A) and (B) are: ◯, PS832 (wt); ⬤, PS832 (wt) heat activated at 70°C for 30 min; △, PS4531 (lacking three YetF homologs); □, PS4461 (wt); and ■, PS4462, PS4461 plus *spoVA*^2mob^ with the *2duf* gene.

Additional factors that can modulate GerA-dependent L-valine germination of *B. subtilis* spores include: (i) heat activation by a short incubation at 70°C, which can increase the rates of L-valine germination by the GerA GR (1, 2, 16), and (ii) spores’ IM fluidity, as (a) L-valine germination is slowed in spores carrying the 2Duf IM protein, which have a less fluid IM than 2Duf-less spores (12, 17), and (b) spores lacking three of the five IM homologs of ~75% of 2Duf (18), which have elevated IM fluidity (19), germinate significantly faster with L-valine than wt spores (Fig. 3A). Notably, although the expected large increase in the rate of L-valine germination was seen in heat-activated wt spores (Fig. 3A), heat activation had no observable effect on the rate of LiCl germination (Fig. 3B; and see Discussion). However, the rate of LiCl germination was increased markedly in spores with increased IM fluidity, whether because of the absence of 2Duf or the absence of multiple homologs of most of 2Duf (Fig. 3B).

**Fig 4:**
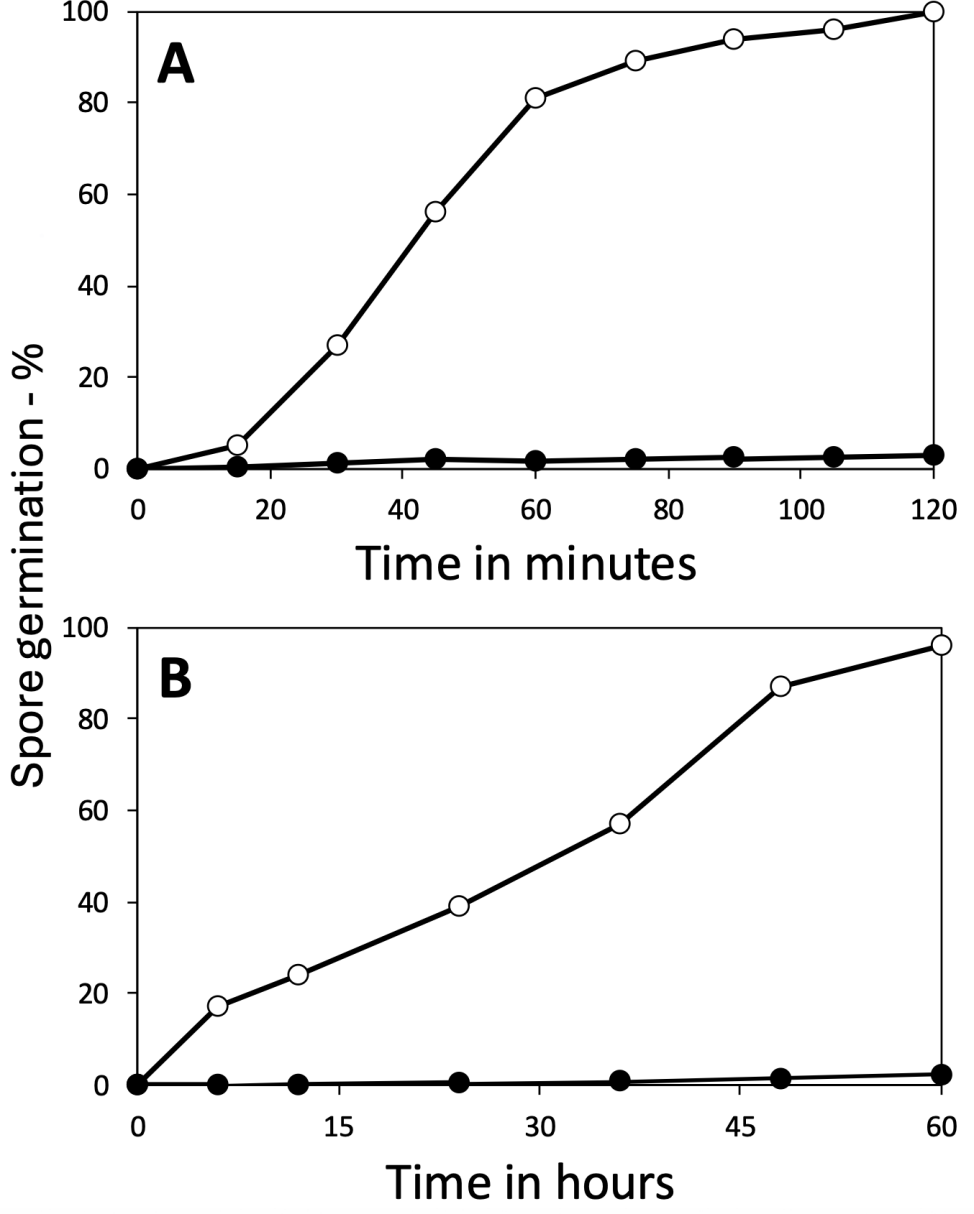
Effects of a *gerAB* mutation on spore germination with (A) L-alanine or (B) LiCl. Spores of *B. subtilis* PY79 with or without the Y97A mutation in *gerAB* were germinated with either (A) L-alanine, or (B) LiCl, and germination was determined by measuring dipicolinic acid as described in Methods. Symbols for the PY79 strains used are: ◯, wt, and ⬤, Y97A. All data are from duplicate germination incubations, and the results were averaged. Variance in individual data points was <8%.

To determine if changes in the likely GerA GR’s germinant binding pocket in a putative water channel in the B subunit (GerAB) (12, 20) were involved in facilitating LiCl germination, a *gerAB* point mutant in the putative water channel in this protein (21) was tested for germination with L-alanine and LiCl (Fig. 4A and B). L-Alanine was the germinant in this experiment because it was the germinant used to test the effects of these GerAB variants on spore germination (21). As was recently reported (21), spores of the GerAB Y97A mutant contained only ~20% of the wt level of the GerAA subunit of the GerA GR. Even so, the GerAB Y97A spores had minimal if any L-alanine germination (Fig. 4A) nor any LiCl germination as well (Fig. 4B). Unfortunately, spores of several other mutants in the putative water channel contained minimal if any GerA GR (21); hence, they were not used in this work.

### Influence of other cations on LiCl germination

**Fig 5:**
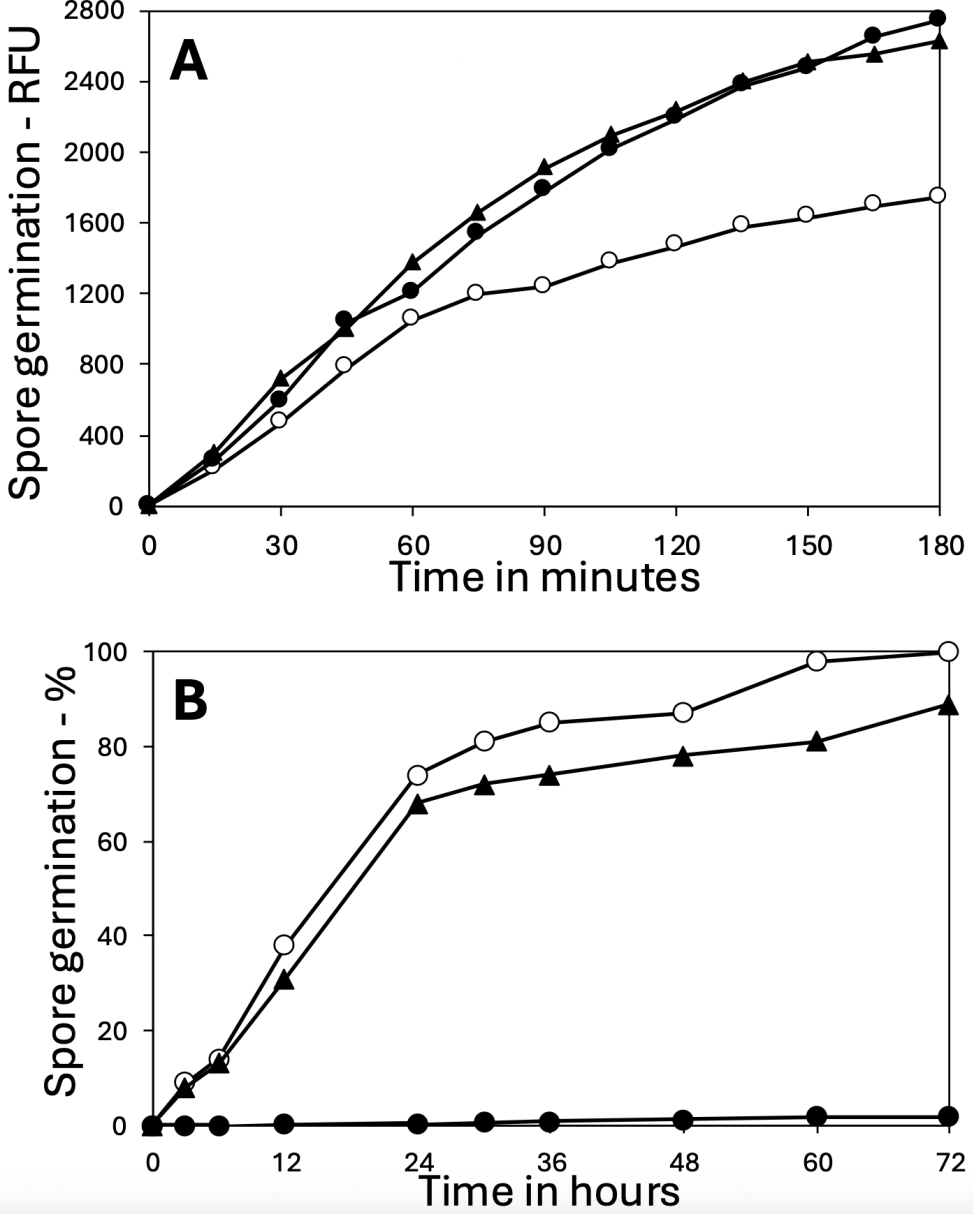
Effects of KCl and NaCl on spore germination with (**A**) L-alanine or (**B**) LiCl. (A) Spores of PS832 were heat activated and germinated in duplicate at 37°C with 10 mM L-alanine in 5 mM K-Hepes buffer (pH 7.4) with or without KCl or NaCl, and germination was measured by Tb-DPA fluorescence as described in Methods, and the results were averaged. Variance in individual data points was <5%. The germination was with no additions (◯), or with 50 mM KCl (▲) or 10 mM NaCl (⬤). (**B**) PS832 spores without heat activation were germinated at 40°C in 100 mM LiCl and 5 mM K-Hepes buffer (pH 7.4), and with no additions (◯), with 10 mM KCl (▲) or 2 mM NaCl (⬤). Germination was determined by measuring dipicolinic acid release as described in Methods. Presented data are average values where variance in individual data points was <10%.

Optimal conditions for spore germination in aqueous suspensions generally include inorganic salts in addition to the organic germinant moiety. In some cases, the germination rate can be influenced by the choice of salt present within the germination buffer. Inosine-mediated germination in *Bacillus cereus* spores, for example, proceeds rapidly in the presence of Na^+^ ions but is strongly inhibited in the presence of K^+^ ions (22). With this in mind, we decided to investigate whether LiCl germination in *B. subtilis* spores was influenced by the presence of Na^+^ or K^+^ ions, and if so, we sought to determine whether the presence of the second cation exerted positive or negative effects on the LiCl germination response. As a control measure, *B. subtilis* PS832 spores were first tested for their germinative response to L-alanine in germination buffer supplemented with either KCl or NaCl, revealing comparable but enhanced germinative rates relative to spores incubated in the non-supplemented germination buffer (Fig. 5A). Similarly, LiCl-mediated germination appeared to proceed efficiently (>80% germination) in buffer supplemented with 10 mM KCl (Fig. 5B). However, NaCl was shown to exert a strong inhibitory effect on LiCl-mediated germination, with less than 2% of the spores germinating in LiCl-containing buffer supplemented with 2 mM NaCl over the observed time period (Fig. 5B).

### LiCl-induced spore germination in other *Bacillus* species

Spores of *B. megaterium* and *B. cereus* were also examined to ascertain whether they too germinated in response to aqueous LiCl in order to provide a pointer as to whether this observation extends across the wider Bacillota family. *B. megaterium* PV361 spores germinated in response to LiCl most efficiently when the GerUV germinant receptor was present, but at a slower rate than observed for *B. subtilis* spores; approximately 16% of PV361 pHT:GerUV spores (GC431) were observed to have germinated after 48 h in the highest concentration of LiCl tested (250 mM), rising to 60% after 8 days (Fig. 1; Table 2). Spores that lacked the GerD protein (GC616) but were otherwise isogenic with strain GC431 showed a reduced rate (30% after 8 days), with further reductions in germinative efficiency in spores lacking either GerUV or all five of the GRs encoded in this species. Notably, the weak germinative response observed in the germinant receptor-less strain (GC614) after 8 days was higher than that observed in the same strain when incubated in 5 mM Tris-HCl buffer alone (17% versus 2%). In general, 250 mM LiCl induced a more efficient germinative response in *B. megaterium* spores than 100 mM LiCl, although a maximal amount of ~60% after 8 days was evident under both conditions. In contrast to *B. subtilis* and *B. megaterium*, spores of *B. cereus* 10876 failed to show any germinative response to 250 mM LiCl over 8 days, regardless of whether spores were heat shocked or not prior to incubation (data not shown).

**TABLE 2:** Germination of *B. megaterium* spores with LiCl^*a*^

Strain	Concentration (mM)^*b*^	Germination (%) after incubation at 30°C for				
		0 h	6 h	24 h	48 h	192 h
PV361 (Δ*gerUV*)	100	0	1	5	6	12
	250	0	1	1	11	9
PV361 pHT-*gerUV*	100	0	2	4	13	60
	250	0	3	6	16	58
PV361 Δ*gerD* pHT-*gerUV*	100	0	3	2	3	30
	250	0	2	1	8	30
PV361 *ger5*	100	0	3	3	6	23
	250	0	4	2	13	17

^*a*^ Spores (~1 × 10^8^ /mL) were incubated at 30°C in 5 mM Tris-HCl (pH 7.5) containing 100 or 250 mM LiCl, and at various times, aliquots were examined by phase contrast microscopy and scored for germination as described in the methods.

^*b*^ Control (buffer only) spore germination was <5% in all cases

## DISCUSSION

The finding that LiCl can trigger spore germination in a GR-mediated response was surprising as LiCl has not previously been found to trigger *Bacillus* spore germination (9, 10). LiCl has been shown to be rapidly taken up in *Bacillus thuringiensis* spores’ outer layers, and even some into the core, but this was not accompanied by the triggering of spore germination (23). In addition, LiCl as well as the salts NaCl, KCl, NH_4_Cl, CsCl, and RbCl have been shown to stimulate the *B. subtilis* GerA response to L-alanine (24), a response that is much more rapid than with LiCl alone seen in the current work. Thus, the germination of *B. subtilis* spores by LiCl alone via GerA activation is a novel finding, as is the triggering of *B. megaterium* spore germination by LiCl, also by activating a GR in this organism. The question then is how LiCl does this, that is, does it directly activate the appropriate GR by action on these GRs’ germinant interaction site or is it a general effect on the particular GR or even on spore IM structure? In this regard, the *B. subtilis* GerA GR is noteworthy, as this GR has been shown to be by far the most sensitive to signals that activate it spontaneously during sporulation, in particular signals that indicate that the sporulation process is not going appropriately (25). GerA-dependent germination via a physiological germinant such as L-valine is also very sensitive to IM fluidity either when: (i) the presence of the IM 2Duf protein decreases IM lipid fluidity, or (ii) the loss of multiple IM YetF homologs increases IM fluidity, all as seen previously (18, 26) and in this work. Notably, the effects of IM lipid fluidity on LiCl germination were essentially identical to the effects on L-valine germination. In addition, the relative effects of the Y97A mutation in GerAB on L-valine and LiCl germination were similar, suggesting that the effects of LiCl are on the GerAB subunit, perhaps facilitating the adoption of a conformation that is more active in triggering germination and subsequent downstream events (4, 27).

**Fig 6:**
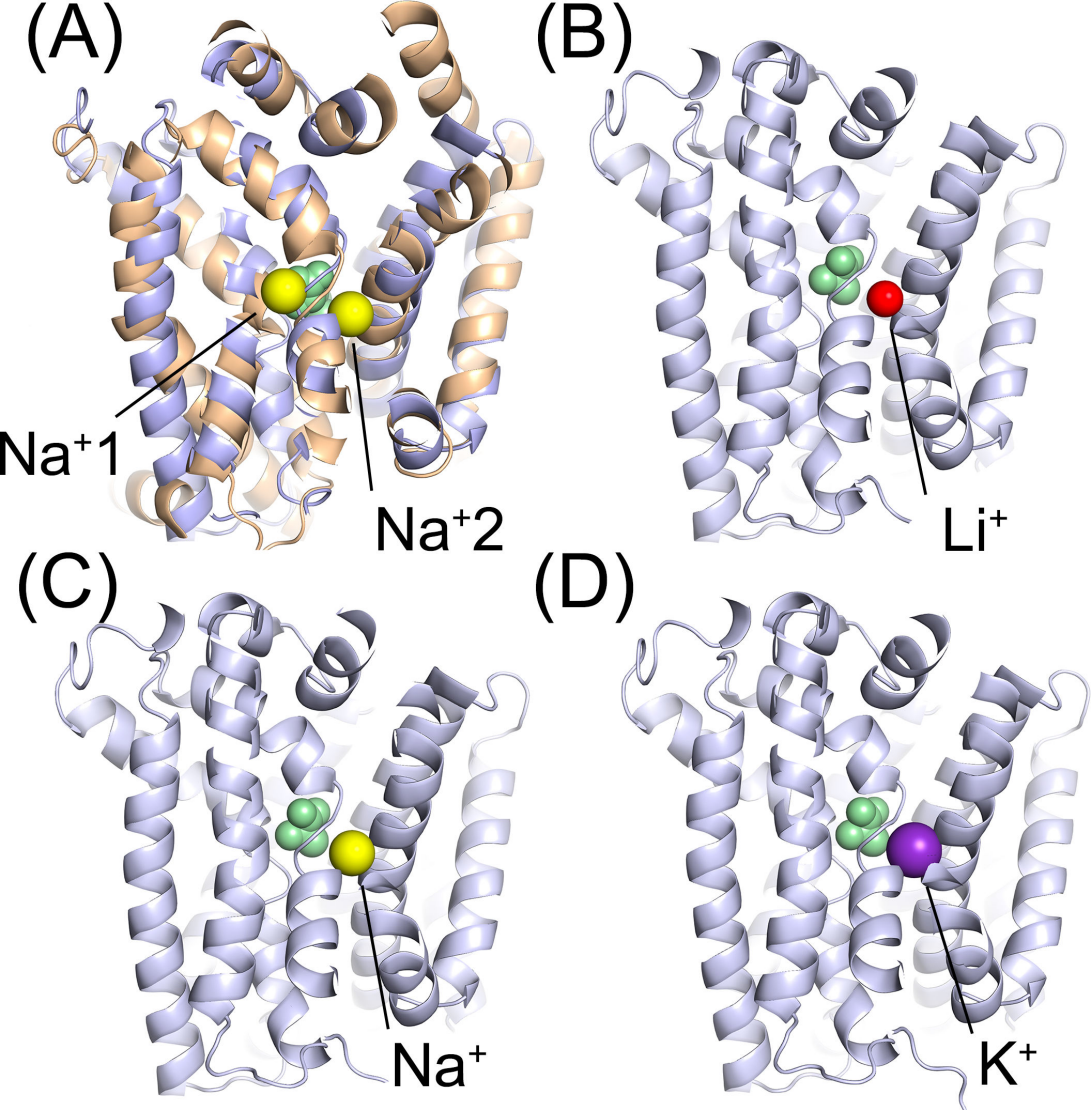
Potential cation binding sites in the *B. subtilis* GerA germinant receptor. (**A**) AlphaFold model of the *B. subtilis* GerAB protein (gray) aligned with the crystal structure of LeuT (light orange; PDB:2A65), a bacterial Na^+^-coupled leucine transporter. Proteins are shown in the plane of the membrane and sliced through to reveal the LeuT leucine substrate (green) and sodium ion (yellow) binding sites. (**B**) – (**D**) Chai-1 structural prediction models of *B. subtilis* GerAB protein with bound L-alanine (green) and Li^+^, Na^+^, and K^+^ ions. The respective cations are predicted to bind in a pocket structurally equivalent to the LeuT Na^+^2 site, regardless of whether the model is generated in the presence or absence of L-alanine.

If GR B-subunit proteins do constitute the interaction site for Li^+^ ions within spores, where precisely might they bind and how might they stimulate germination in the absence of cognate organic germinant molecules? Why also is it only certain GRs that appear to be stimulated by Li^+^ ions to trigger the germination cascade? The availability of AlphaFold models of GR B-subunit proteins and crystal structures of secondary transporter proteins from the wider APC superfamily to which GR B-subunits belong confers a structural basis to address these questions. For example, AlphaFold models of spore GR B-subunit proteins align closely when superposed with crystal structures of numerous APC transporters, sharing the 5-helix inverted repeat core structure that is characteristic of this family (28). A second feature common to many APC transporters concerns the symport of monovalent cations, typically sodium, in order to facilitate the transport of cognate organic ligands. In these cases, electrochemical gradients established across the membrane satisfy the thermodynamic requirements associated with conformational changes that drive the transport of ligands. The bacterial leucine transporter, LeuT_Aa_, for example, has two Na^+^ binding sites located in close proximity to the leucine binding site that is positioned toward the middle of the protein (29). One of these ion binding sites, so-called Na^+^2, is conserved in crystal structures of other transporters, including Mhp1 and vSGLT (28). Structural alignment of *B. subtilis* GerAB with ligand and ion-bound LeuT_Aa_ places both Na^+^ ions close to unwound helical domains that are central to ligand binding and function of all APC-type proteins (Fig. 6). Notably, LeuT_Aa_’s bound leucine moiety occupies the same space that L-alanine is predicted to occupy in GerAB (12). It seems plausible then, given the importance of cations in influencing GR-mediated spore germination, that positions within GR B-subunit proteins analogous to LeuT_Aa_ Na^+^1 and/or Na^+^2 may serve as cation binding sites that potentiate binding of cognate germinant molecules. Indeed, site-directed mutagenesis experiments conducted on the B-subunit of the *B. cereus* GerI GR indicate that residues in the vicinity of the Na^+^2 location within GerIB are extremely sensitive to modification, with some substitutions completely abolishing inosine-mediated spore germination (unpublished results). In a similar vein, the inhibitory effect of certain cations on GerI-mediated germination (22) was mirrored in the current work, albeit in this case, Na^+^ ions were inhibitory to the *B. subtilis* GerA-mediated LiCl response, whereas K^+^ ions exerted no apparent effect.

Collectively then, data from inhibition assays coupled with structural modeling-derived observations suggest that different monovalent cations are competing for binding at a common cation binding site within GR B-subunit proteins, and most likely at positions analogous to cation binding sites present in APC transporters. However, even if this is the case, it is not immediately apparent why the binding of Li^+^ but not other monovalent cations triggers germination in some spore GRs but not others. Differences in the atomic radii of monovalent cations may be important, that is, the Li^+^ ion is smaller than Na^+^, which in turn is smaller than K^+^; hence, perhaps subtle structural differences in GR B-subunit proteins mean that Li^+^ can bind in regions that are inaccessible to larger cations, somehow triggering germination. Data reported in this work, for example, support models where (i) GerAB and GerVB have Li^+^ binding pockets but GerIB does not, and (ii) Na^+^ can block access to the Li^+^ binding pocket in GerAB, whereas larger K^+^ ions are ineffective in doing so.

The nature of the cation interaction with receptor protein and subsequent transduction or dissipation of free energy must also be considered, particularly if the thermodynamic basis for cation-associated germination is to be established. A precedent for this can be found in the wider APC superfamily of proteins, where the specificity and activity of certain metal ion transporters are determined by disparities in binding energy associated with different cations (30) and perhaps similar principles apply to spore germinant receptor proteins. In this regard, the observation that heat activation appeared not to influence the rate of LiCl-associated germination, in contrast to the L-valine response, may be telling. One explanation for the lack of heat activation effect may be suggested by the fact that heat activation is reversible, a process that is faster at higher temperatures such as 40°C (31). However, since LiCl germination at 40°C is slow, the reversal of heat activation during LiCl germination itself may greatly decrease the effects of the initial heat activation on this germination. However, from a thermodynamic perspective, changes in free energy upon Li^+^ ion binding to the GR B-subunit protein may nullify the effects of heat activation, that is, if the binding is endothermic, then this could essentially cool and reverse presumed conformational changes associated with heat activation, resulting in a slow germination process. Similarly, if Li^+^ binding is exothermic, then this may render redundant any rate benefits conferred by the heat activation process, the dissipation of free energy upon binding providing the extra stimulus that initiates the germination process, albeit slowly, in spores with GRs that are cognate for Li^+^ ions.

A number of factors then, the absence of high-resolution experimental structures of any GR B-subunit proteins, a largely incomplete picture of what “heat activation” entails at the molecular level, and minimal insight into the thermodynamics of ion or germinant binding, mean that we are unable to answer any of these questions with certainty at present, but they do provide clear avenues for future work in this area. Ultimately, what appears at first as a biological curiosity in the field of spore germination, happened upon chance observation, actually invokes deeply fundamental questions concerning the underlying principles of the process. The challenge moving forward, as ever with these most intractable bacterial structures, is how best to address these.

## MATERIALS AND METHODS

### Strains and spore preparation and purification

The *Bacillus* strains and species used in this work are listed in Table 3, as are the specific genotypes. Some *B. subtilis* strains had been made previously and are isogenic with the wild-type (wt) 168 strain, PS832; two other *B. subtilis* strains, PS4461 and PS4462, were described recently (26) and are isogenic only with each other; some additional *B. subtilis* strains are isogenic with strain PY79 (21). In some cases, new strains of PS832 were made using DNA from strains carrying an antibiotic resistance marker replacing the gene(s) of interest and additional strains were made in which the antibiotic marker was removed using the Cre recombinase, all as described (32). Note that for the majority of the strains generated and used in this work, their genotype was validated by sequencing and subsequent analyses of the mutant strains’ entire genomes.

**TABLE 3:** *Bacillus* species and strains used

Species	Strain	Antibiotic marker	Relevant genotype	Source (ref)
*B. subtilis*	PS832	None	wild-type (wt)	Laboratory strain
*B. subtilis*	PS4461	None	Wt (not PS832 background)	(26)
*B. subtilis*	PS4462	None	PS4461 with *spoVA*^2mob^ and *2duf*	(26)
*B. subtilis*	PS4498	Erm	Lacks all 5 GR operons	This work
*B. subtilis*	PS4531	Erm	Lacks YrbG, YdfR, YkjA, YdfS	(19)
*B. subtilis*	FB62	Spc	Lacks GerD	(33)
*B. subtilis*	PS4492	Km	Lacks GerA	This work
*B. subtilis*	PS4584	Erm	Lacks GerK	This work
*B. subtilis*	PS4585	Erm	Lacks GerB	This work
*B. subtilis*	PS4586	Km	Lacks YndD	This work
*B. subtilis*	PS4587	Erm	Lacks YfkQ	This work
*B. subtilis*	PS4589^*b*^	None	Lacks GerA^*a*^	This work
*B. subtilis*	PS4590^*b*^	None	Lacks GerK^*a*^	This work
*B. subtilis*	PS4591^*b*^	None	Lacks GerB^*a*^	This work
*B. subtilis*	PS4592^*b*^	None	Lacks YndD^*a*^	This work
*B. subtilis*	PS4593^*b*^	None	Lacks YfkQ^*a*^	This work
*B. subtilis*	PY79	None	wt	Chen
*B. subtilis*	PY79	Erm	GerAB-G25A	(21)
*B. subtilis*	PY79	Erm	GerAB-Y97A	(21)
*B. subtilis*	PY79	Erm	GerAB-L199A	(21)
*B. subtilis*	PY79	Erm	GerAB-G200A	(21)
*B. subtilis*	PY79	Erm	GerAB-F342A	(21)
*B. subtilis*	PY79	Erm	GerAB-triA	(21)
*B. cereus*	10876	None	wt	Anne Moir
*B. megaterium*	PV361	None	plasmidless derivative of QM B1551 wt strain	Pat Vary
*B. megaterium*	GC431	Erm	pHT-*gerUV*	(34)
*B. megaterium*	GC617	Km	lacks GerD; pHT-*gerUV*	(34)
*B. megaterium*	GC614	Km Erm Cm Spc	lacks all 5 GR operons (*ger5*)	(34)

^*a*^ The abbreviations for antibiotic resistance markers are: Erm, erythromycin (Em^r^), 5 mg/mL; kanamycin (Km^r^), 10–20 mg/mL; and spectinomycin (Sp^r^), 100 mg/mL.

^*b*^ These strains were originally made by replacing the genes’ coding regions with an antibiotic resistance marker, and then, the antibiotic marker was removed by the Cre recombinase.

In most experiments, spores of *B. subtilis* strains were prepared on double strength Schaeffer’s-glucose (2xSG) medium agar plates (35) that were incubated for 3–4 days at 37°C. The spores were then scraped from the plates and subjected to repeated washes by centrifugation with intermittent sonication, centrifuged through ~50% Histodenz in which spores pellet and cells and debris float, and finally, Histodenz was removed by washing with water, all as described (36). *B. megaterium* spores were prepared in supplemented nutrient broth (SNB) in a shaking (225 rpm) incubator for 72 h at 30°C and harvested and purified as described previously (34). *B. cereus* spores were prepared on SNB plates at 30°C as described (37), and spores were purified by repeated washes by centrifugation, but without sonication to avoid damaging these spores’ exosporium, with a final step again being centrifugation through Histodenz. All spores used in this work were >97% free of growing cells, germinated spores, or obvious cell debris as determined by examination using phase contrast microscopy. Spores were stored at 4°C in water at an optical density at 600 nm (OD_600_) of ~10 (~ 10^9^ spores/mL) and protected from light.

### Measurement of spore germination

Unless noted otherwise, *B. subtilis* spore germination used spores at an optical density of 600 nm (OD_600_) of 2–5 that were heat activated in water at 70°C for 30 min, cooled on ice, and used within 30–60 min. In some experiments, spores of *B. subtilis* at an OD_600_ of ~0.5 (~ 10^8^ spores/mL) were germinated at 40°C in 200 µL with 10 mM L-valine or L-alanine, both GerA GR-dependent germinants, and in 25 mM K-Hepes buffer, pH 7.4, plus ~50 µM TbCl_3_, and Tb-DPA fluorescence was measured in duplicate at various times in a fluorometric plate reader as described (38). Germination with LiCl was at 40°C and also in 25 mM K-Hepes buffer, pH 7.4, and was much slower than L-valine germination and therefore was routinely monitored by either: (i) phase contrast microscopy of ≥100 spores and determining the percentages of spores that had changed from phase bright (dormant) to phase dark (fully germinated); or (ii) determining the percentage of DPA release at various times by centrifuging ~0.5 mL samples of incubations, saving the supernatant fluid, adding 0.5 mL water to the pellet, boiling for 30 min, centrifuging, and again saving the supernatant fluid. DPA in 5, 10, and 25 mL aliquots of the two supernatants were measured in duplicate in a total of 200 mL of 25 mM K-Hepes buffer pH 7.4 and 50 mM TbCl_3_, and DPA fluorescence was measured in a fluorometer. In one experiment, the effects of various concentrations of NaCl or KCl on spore germination at 40°C in 5 mM K-Hepes buffer pH 7.4 with 100 mM LiCl or L-alanine was tested.

For *B. megaterium,* germination data were collected principally via phase contrast microscopy analyses since absorbance measurements at 600 nm were compromised by clumping of spores over the duration of the experiment. Concentrated *B. megaterium* spore suspensions (OD_600_ ~50) were heat activated at either 60°C for 10 min or 70°C for 30 min, depending on the strain being examined, and then cooled on ice. Spores were suspended at an OD_600_ of ~0.4 (~10^8^ spores/mL) in 5 mM Tris-HCl buffer, pH 7.5, containing either 100 mM or 250 mM LiCl in a final volume of 200 µL in film-sealed 96-well plates and incubated at 30°C. Germination data were collected by analyzing phase contrast microscopy images of spore samples collected in triplicate at defined time points over 8 days. Image analysis was automated to monitor an average of ~1,500 spores per strain per time point, using an ImageJ-based macro (analyze >3d objects counter >threshold 4,095 > size filter 20–180 pixels > exclude objects on edges > maps to show: objects > results tables to show: summary). The program calculated the total number of phase bright spores within the field of view with germinated (phase dark) spores being counted manually in order to determine the proportion of germinated spores at defined time points. An identical experimental procedure was used to examine the effect of LiCl on *B. cereus* 10876 spores.

### Protein modeling

Structural models of germinant receptor proteins were generated using AlphaFold 3 (39). The Chai-1 structural prediction tool (40) was used to predict L-alanine and cation binding sites in the GerAB protein. Structural alignments and rendering of molecules were conducted with PyMOL version 3.03 (Schrödinger, Inc.).
